# Fibronectin mediates activation of stromal fibroblasts by SPARC in endometrial cancer cells

**DOI:** 10.1186/s12885-021-07875-9

**Published:** 2021-02-12

**Authors:** Sachiko Yoshida, Kazuo Asanoma, Hiroshi Yagi, Ichiro Onoyama, Emiko Hori, Yumiko Matsumura, Kaoru Okugawa, Hideaki Yahata, Kiyoko Kato

**Affiliations:** Department of Obstetrics and Gynecology, Faculty of Medical Sciences, Kyushu University, 3-1-1 Maidashi, Higashi-ku, Fukuoka, 812-8582 Japan

**Keywords:** Cancer-associated fibroblasts, Endometrial neoplasms, Extracellular matrix, FN1, SPARC

## Abstract

**Background:**

Matricellular glycoprotein, SPARC is a secreted molecule, that mediates the interaction between cells and extracellular matrix. SPARC functions as a regulator of matrix organization and modulates cell behavior. In various kinds of cancer, strong SPARC expression was observed in stromal tissues as well as in cancer epithelial cells. The function of SPARC in cancer cells is somewhat controversial and its impact on peritumoral stromal cells remains to be resolved.

**Methods:**

We investigated the effects of SPARC expression in endometrial cancer cells on the surrounding stromal fibroblasts using in vitro co-culture system. Changes in characteristics of fibroblasts were examined by analysis of fibroblast-specific markers and in vitro contraction assay.

**Results:**

SPARC induced AKT phosphorylation and epithelial-to-mesenchymal transition, consistent with previous reports. Cancer-associated fibroblasts of endometrial cancer expressed higher levels of mesenchymal- and fibroblast-associated factors and had a stronger contraction ability. Unexpectedly, cancer-associated fibroblasts expressed comparable levels of SPARC compared with fibroblasts from normal endometrium. However, co-culture of normal fibroblasts with SPARC-expressing Ishikawa cells resulted in activation of the fibroblasts. Immunodepletion of SPARC did not affect the activation of fibroblasts.

**Conclusions:**

Our data indicated that SPARC activated fibroblasts only in the presence of fibronectin, which was abundantly secreted from SPARC-expressing endometrial cancer cells. These results suggested that a SPARC-fibronectin-mediated activation of fibroblasts might be involved in enhanced mobility and invasion of cancer cells.

**Supplementary Information:**

The online version contains supplementary material available at 10.1186/s12885-021-07875-9.

## Background

The matricellular glycoprotein, SPARC is a secreted molecule that mediates interactions between the cell and extracellular matrix. SPARC functions as a regulator of matrix organization and modulates cell behavior [1, 2]. Various functions of SPARC in cancer cells have been reported. In addition to modulation of extracellular matrix, SPARC also regulates cell adhesion, proliferation, survival, apoptosis, migration, invasion, and induction of epithelial-to-mesenchymal transition (EMT) in cancer cells [1, 3–5].

In addition to functions in cancer cells, SPARC also plays a critical role in stromal cells in in cancer progression. In various kinds of cancer, strong SPARC expression was observed in stromal cells in contrast with its low expression in cancer epithelial cells [6–9]. While SPARC secreted from stromal fibroblasts was suggested to suppress cancer cell proliferation or migration in vitro, SPARC expression in peritumoral fibroblasts correlated with worse prognosis in pancreatic cancer [8–10].

The effects of SPARC in host tissues have been studied by transplantation of cancer cells into SPARC-deficient mice. While murine mammary cancer cells transplanted in SPARC-deficient mice formed smaller tumors compared with controls, murine pancreatic cancer cells, Lewis lung cancer cells and lymphoma cells formed larger tumors and increased metastasis in the mice [11–15]. Mouse carcinogenesis models in a SPARC-deficient background have been also studied. Prostate and bladder carcinogenesis is enhanced in SPARC-deficient mice [16, 17]. However, other studies showed that skin squamous cell carcinoma and intestinal tumors were suppressed in SPARC-deficient mice [18, 19]. Another report of SPARC-deficient mice did not find any changes in the cancer progression and metastasis in prostate and mammary carcinogenesis [20].

Therefore, the function of SPARC in oncogenesis is somewhat controversial and it cannot be determined based only on the endogenous expression of SPARC in cancer cells. Other factors including interactions with tumor microenvironment including extracellular matrix, stromal cells or proteolysis of SPARC may be involved but the mechanism remains largely unknown [1, 21–23].

In our previous study, we found that SPARC was exclusively expressed in endometrial cancer (EC) stem-like cells. EC cells with forced expression of SPARC formed tumors with larger amounts of peritumoral stromal tissue after subcutaneous inoculation in nude mice [24]. We also observed that while SPARC expression rate was low (28%) in well differentiated endometrioid carcinoma grade 1 cases, its expression was enhanced in poorly differentiated endometrioid carcinoma, grade 3 (60%), serous carcinoma (50%) and clear cell carcinoma cases (73%), which were thought to have aggressiveness [24]. However, the impact of the SPARC expression on the association between tumor cells and the adjacent stromal cells has not been well determined.

## Methods

### Fibroblast isolation and cell culture

Normal fibroblasts (NF) were isolated from six patients who underwent fertility treatment at the IVF Nagata Clinic (Table S1) [25]. Endometrial tissues were digested with 0.25% collagenase type I solution (Thermo Fisher Scientific, Inc., Waltham, MA, USA) for 2 h at 37 °C using a rotating mixer. The cells were passed through 35 μm cell strainer (Corning, Kennebunk, ME, USA), and suspended in Dulbecco’s modified Eagle’s medium (DMEM, Nacalai Tesque, Kyoto, Japan) supplemented with 10% fetal bovine serum (FBS; Merck KGaA, Darmstadt, Germany), and 1% penicillin/streptomycin (Nacalai Tesque). The cells were cultured at 37 °C at 5% CO_2_ atmosphere. Cancer associated fibroblasts (CAF) were isolated from seven patients who underwent surgery at the Department of Obstetrics and Gynecology at Kyushu University Hospital (Table S2). Cancer tissues were minced and digested 2 mg/mL collagenase A (100 mg/ml, Sigma Aldrich, St. Louis, MO, USA) with DMEM containing 10% FBS for 20 min at 37 °C. The cells were passed through a 40-μm cell strainer (Corning) and cultured in DMEM with 10% FBS and 1% penicillin/streptomycin. NF and CAF at passage 3¬8 were used for experiments as were chosen also in other studies [26–31]. Written informed consent was obtained from all patients. The study was approved by the Ethical Committee of Kyushu University. Ishikawa EC cells were purchased from Japanese Collection of Research Bioresources (JCRB, Tokyo, Japan), and maintained in DMEM supplemented with 10% FBS and 1% penicillin/streptomycin. Ishikawa cells were used within 6 months of receipt.

### Reagents

The AKT inhibitor, MK2206 (Selleck Chemicals, Houston, TX, USA) was used on Ishikawa cells at the indicated concentrations. Cells treated with MK2206 for 24 h were used for immunoblotting. For migration assays, MK2206 was added to both the upper and the lower chambers. Recombinant human SPARC was purchased from PeproTec Inc. (Rocky Hill, NJ, USA). One hundred ng/mL of recombinant SPARC was used on fibroblasts unless otherwise indicated. Fibronectin (FN1) was purchased from Corning, and plates were coated with FN1 at a concentration of 50 μg/mL.

### Lentivirus vector transduction

FLAG-tagged SPARC plasmid was kindly provided by Dr. Sasaki Takako at Oita University, and was ligated into a pLX302 vector. An empty pLX302 vector was used as a control. Lentivirus vectors were produced as described previously [32]. To generate stable cell lines, lentivirus-infected Ishikawa cells were cultured in 2 μg/mL of puromycin.

### Collagen gel contraction assay

Collagen lattices were prepared using Cellmatrix (Nitta Gelatin Co., Osaka, Japan), sterile reconstitution buffer (50 mM NaOH, 2.2% NaHCO_3_, 200 mM HEPES) and 5 × DMEM (Nissui Pharmaceutical Co., Tokyo, Japan) mixed at a ratio of 7:2:1. NF were suspended in the collagen solution (2 × 10^5^ cells/ml). Next 300 μL of the mixture was poured into 24-wells plates, and the mixture was allowed to polymerize for 30 min at 37 °C. After polymerization, 300 μL serum-free DMEM was added to the collagen gel. After 24 h, the collagen gel was released mechanically and the area of the gel was measured 24 h later. The contraction ratio (%) was calculated as (well area − gel area) / (well area) × 100.

### Immunohistochemistry

Paraffin sections were deparaffinized, and rehydrated. The slides were washed in PBS after applying Proteinase K (Dako, Carpinteria, CA, USA) for 6 min and blocked in Protein Block Serum Free (DAKO) for 30 min at room temperature. The slides were immunolabeled with primary antibodies against SPARC (1:500, M125, TAKARA, Tokyo, Japan) and FN1 (1:300, ab2413, Abcam, Cambridge, UK) overnight at 4 °C after incubation with Antibody Diluent Solution (DAKO). The slides were then rinsed with PBS and incubated with N-Histofine Simple Stain MAX PO (Nichirei, Tokyo, Japan) at room temperature for 30 min. Visualization of the immunoreaction was carried out by incubation with 3,3-diaminobenzidine (DAB). Finally, sections were counterstained with hematoxylin. Images were captured using Olympus Microscope BX53 and cellSens imaging software (Olympus, Tokyo, Japan). The images were acquired at a resolution of 600dpi.

### Immunocytochemistry

Fibroblasts were seeded into 8-well Lab TekII chamber slides (Thermo Fisher Scientific) at a density of 1 × 10^5^ cells per chamber. After 24 h incubation, the cells were fixed in 4% paraformaldehyde for 15 min, permeabilized with 0.25% Triton − 100 in PBS for 10 min, and blocked with Protein Block Serum Free (Dako) for 10 min at room temperature. The cells were incubated at 4 °C overnight with primary antibodies against VIM (1:200, sc-6260, Santa Cruz Biotechnology, Santa Cruz, CA, USA) and pan-Cytokeratin (1:200, sc-8018, Santa Cruz Biotechnology). The cells were incubated at room temperature for 30 min with a secondary antibody, Alexa Fluor 488 goat anti-mouse IgG (1:500, Life Technologies, Waltham, MA, USA). Cells were mounted in ProLong Gold antifade reagent with 4′,6-diamidino-2-phenylindole (DAPI) (Invitrogen, Carlsbad, CA, USA) and visualized by BZ-X710(KEYENCE Corporation, Osaka, Japan).

### Immunoblotting

Cells were lysed in CelLyticM (Sigma Aldrich) with protease inhibitor cocktail (Sigma Aldrich) and phosphatase inhibitor cocktail (Nacalai Tesque). Equal amounts of lysate were separated by 10% sodium dodecyl sulfate polyacrylamide gel electrophoresis (SDS-PAGE) and transferred to a polyvinylidene difluoride membrane (Millipore, Bedford, MA, USA). The membranes were blocked with 5% milk or Blocking One-P (Nacalai Tesque) for 20 min, and then incubated at 4 °C overnight with primary antibodies against ACTA2 (1:1000, A2547, Sigma-Aldrich), FAP (1:500; ab28244, Abcam), VIM (1:1000, sc-6260, Santa Cruz Biotechnology), CDH2 (1:1000, sc-7939, Santa Cruz Biotechnology), CDH1 (1:1000, #3195, Cell Signaling Technology, Beverly, MA, USA), SPARC (0.75 μl/mL, AON-5031, Haematologic Technologies Inc., Essex Junction, VT, USA), FN1 (1:1000, ab23750, Abcam), GAPDH (1:1000, sc-25,778, Santa Cruz Biotechnology), ACTB (1:1000, sc-81,178, Santa Cruz Biotechnology), FLAG M5 (1:5000, F4042, Sigma-Aldrich), phosphorylated-AKT (Ser473, 1:1000, #9271, Cell Signaling Technology) and AKT (1:1000, #9272, Cell Signaling Technology). The membranes were incubated at room temperature for 1 h with the appropriate secondary antibody, mouse or rabbit IgG-Peroxidase (Sigma-Aldrich). The blots were processed using enhanced chemiluminescence and bands were detected using a ChemiDoc XRS Imaging System (Bio Rad, Richmond, CA, USA).

### Elisa

After NF and Ishikawa cells were incubated with serum-free DMEM for 24 h, the culture medium was collected. The concentrations of SPARC and FN1 were analyzed using enzyme-linked immunosorbent assays (SPARC, DSP00; FN1, DFBN10, R&D Systems, Minneapolis, MN, USA) according to the manufacturer’s instructions.

### Real-time reverse transcription polymerase chain reaction (RT-qPCR)

Total RNA was extracted from cells using RNeasy Mini Kits (Qiagen, Valencia, CA, USA), and cDNA was generated by ReverTra Ace-α (Toyobo, Osaka, Japan). Real-Time RT-PCR was performed using TaqMan primers and primers for SPARC (Hs00234160_m1), FN1 (Hs00365052_m1), VIM (Hs00185584_m1), CDH2 (Hs00983056_m1), and CDH1 (Hs01023894_m1) provided by Thermo Fisher Scientific (Waltham, MA, USA). qPCR was performed using TaqMan Fast Advanced Master Mix and StepOnePlus system (Thermo Fisher Scientific). Gene expression levels were obtained using individual values normalized to 18S rRNA (Hs99999901_s1, Thermo Fisher Scientific).

### Migration assay

Cells (2.5 × 10^5^) suspended in serum-free medium were plated on a Falcon cell culture insert with 8.0 μm pores (Corning). DMEM with 10% FBS was placed in the lower wells. After 48 h, the membranes were collected and stained with Diff-Quick staining solutions (Sysmex Corp., Kobe, Japan).

### Knockdown of FN1 gene expression in Ishikawa cells

A validated short interfering RNA for FN1 (siFN1; siGENOME Human FN1(2335) siRNA-SMART pool) and negative control siRNA (siGENOME Non-Targeting siRNA pool#2: Dharmacon) were transfected into SPARC-expressing Ishikawa cells using Lipofectamine 2000 (Thermo Fisher Scientific). Forty-eight hours after transfection, the cells were used for immunoblotting or cultured in serum-free medium for another 48 h for production of conditioned medium.

### Immunodepletion of FLAG-tagged SPARC in conditioned media

Conditioned media from Ishikawa cells were incubated with anti-FLAG M2 affinity gel (1:300) at 4 °C with agitation overnight. The media were centrifuged at 5000 g for 2 min to remove debris.

### Co-culture of NF with Ishikawa cells

NF were co-cultured with Ishikawa cells in a Falcon cell culture insert with 0.4 μm pores (Corning, Kennebunk, M). For collagen gel contraction assay, NF were collected after 24 h co-culture with Ishikawa cells and used for assays.

### Statistical analysis

Data are represented as the means ± standard deviation (SD) and were analyzed with two-sided Student’s *t*-test. The data in Figs. 5d, e, g, h and 6c, d, g and h were analyzed with one-way ANOVA (analysis of variance) followed by Newman-Keuls test. *P*-values of less than 0.05 were considered statistically significant.

## Results

### SPARC expression in NF and CAF from EC

**Fig. 1:**
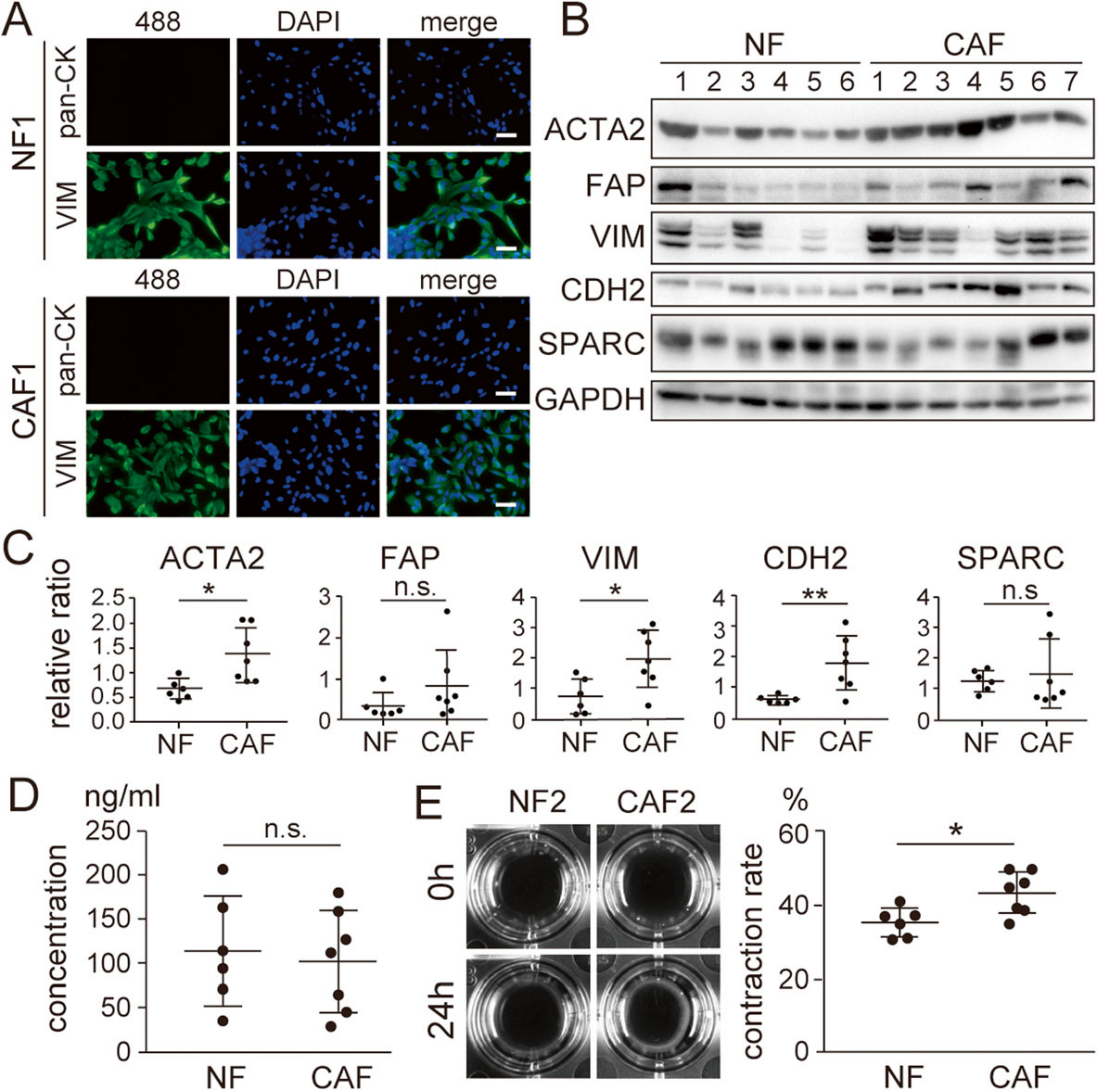
**Characteristics of isolated fibroblasts from normal endometrium and endometrial cancer specimens.** (A) Fibroblasts isolated from normal endometrium sample #1 (top, NF1) and those from endometrial cancer sample #1 (bottom, CAF1) were used for immunocytochemistry. Anti-pan-cytokeratin (pan-CK) and anti-VIM antibodies were used to visualize protein expression. (B) Isolated NF (*n* = 6) and CAF lines (*n* = 7) were used for immunoblotting to detect the expression of ACTA2, FAP, VIM, CDH2 and SPARC. GAPDH was used as an internal control. Full-length blot images are presented in Supplementary Fig. 1. (C) Semi-quantification of the immunoblotting data in (B). Intensity of the bands was quantified using Image J (https://imagej.nih.gov/ij/). Values of the protein-of-interest were corrected using the intensity of GAPDH bands. (D) ELISA analysis of SPARC secreted from the NF and CAF lines. (E) In vitro contraction analysis of the NF and CAF lines. The images show representative results of the experiments. The graphs on the right show quantification data of the results. The scale bars indicate 100 μm. 488, Alexa Fluor 488; pan-CK, pan-cytokeratin; NF, normal fibroblasts; CAF, cancer-associated fibroblasts; n.s., not significant; *, *P* < 0.05

Cancer cells are closely associated with the surrounding stromal fibroblasts. A specific type of activated fibroblasts, CAF plays a critical role in cancer cells in creating the extracellular matrix and tumor microenvironment [33]. In our previous study, we found that abundant SPARC protein localized in tumor stroma is associated with an aggressive type of EC [24]. To explore the impact of SPARC expression in CAF, we first compared the characteristics between NF from normal endometrium and CAF isolated from EC specimens. Successful isolation of fibroblasts from normal endometrium and EC specimens was confirmed by immunocytochemistry (Fig. 1a). All cells were positive for VIM expression and negative for pan-cytokeratin expression. As reported elsewhere [33], higher expression of markers, such as ACTA2, VIM and CDH2 for activated fibroblasts, was observed in CAF from EC compared with NF (Fig. 1b and c; Supplementary Fig. 1). Unexpectedly, the expression and secretion of SPARC was comparable between NF and CAF (Fig. 1b-d; Supplementary Fig. 1). We also evaluated the functional activity of fibroblasts by a collagen gel contraction assay. As expected, CAF showed a higher contraction rate than NF (Fig. 1e). These results suggested that the abundant SPARC protein localized in the stroma of aggressive EC specimens was not produced by the stromal fibroblasts.

### Forced expression of SPARC in EC cells enhanced EMT and cell mobility mediated by AKT activation

**Fig. 2:**
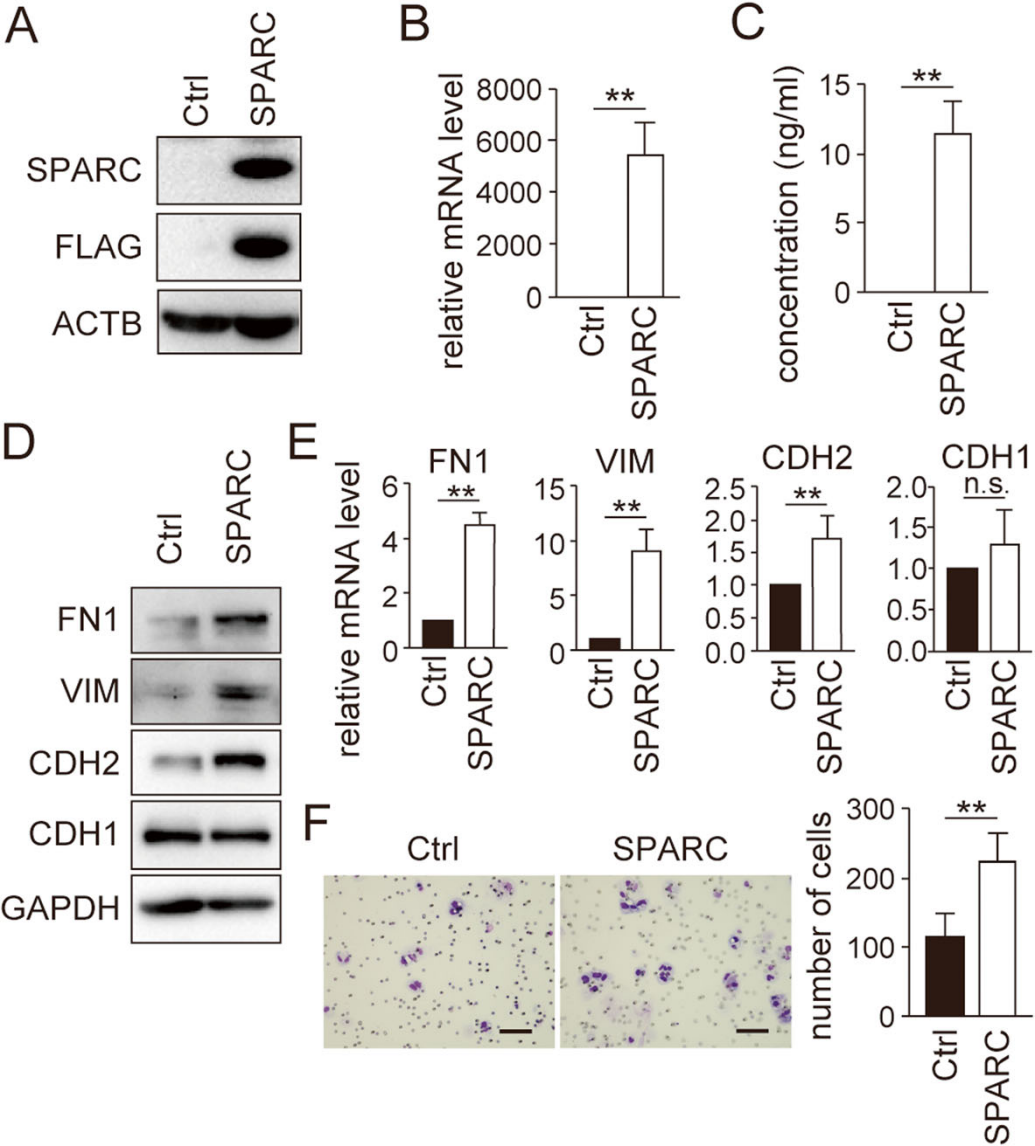
**Forced expression of SPARC in Ishikawa cells induces EMT and cell migration.** Successful lentiviral transduction of SPARC in Ishikawa cells was confirmed using immunoblotting (A), RT-qPCR (B) and ELISA (C). ACTB was used as an internal control (A). (D) The expression of FN1, VIM, CDH2 and CDH1 protein and mRNA in SPARC-expressing Ishikawa cells was analyzed using immunoblotting (D) and RT-qPCR (E). (F) In vitro cell migration was analyzed using transwell chamber assays. Images on the left show representative results. Graphs on the right show quantification data of the results. The scale bars indicate 100 μm. Ctrl, control; n.s., not significant; *, *P* < 0.05; **, *P* < 0.01. Full-length blot images are presented in Supplementary Fig. 2 and 3 (A, D). The experiments were independently repeated three times and representative data were shown

**Fig. 3:**
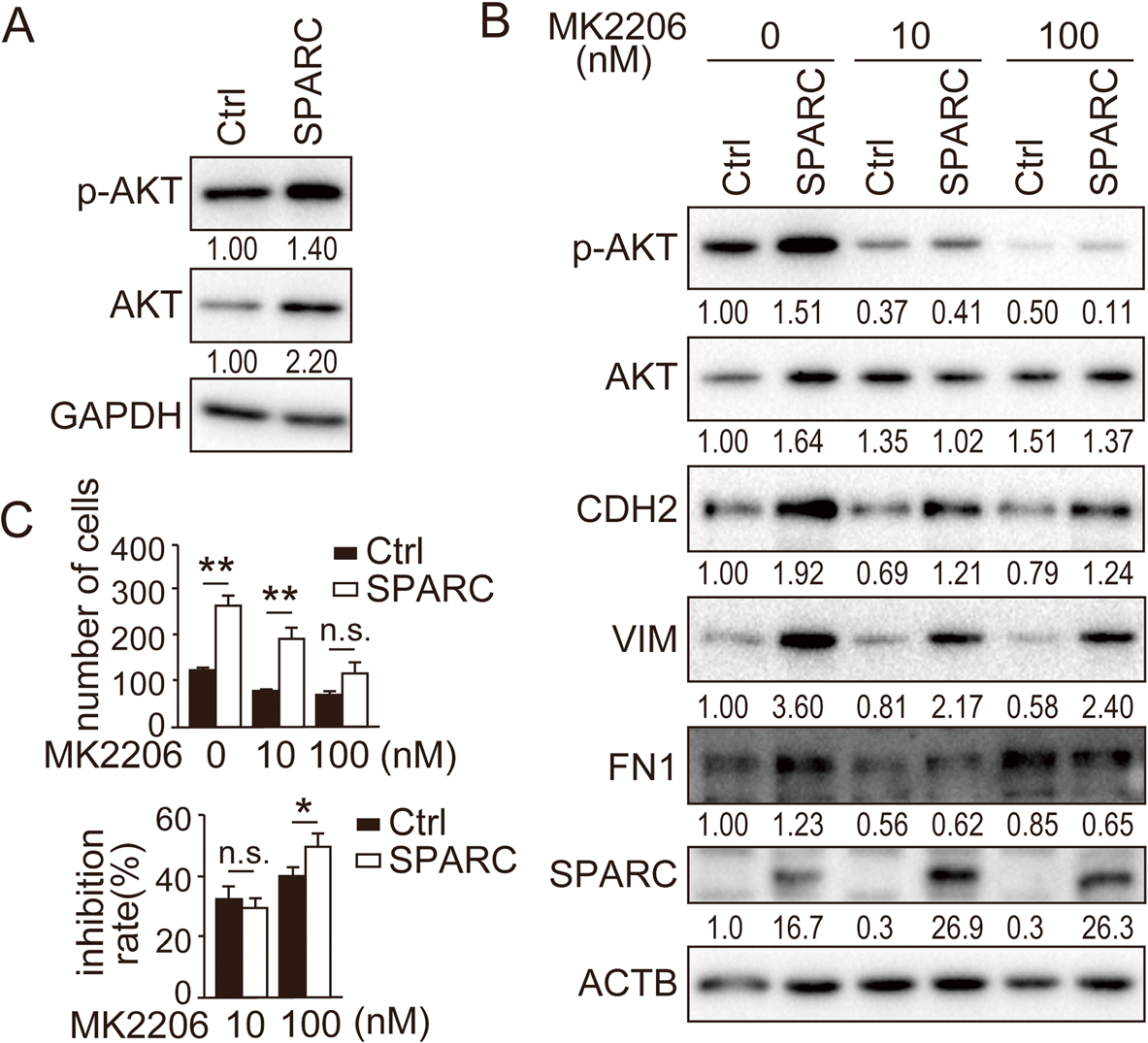
**Induction of EMT and cell migration by SPARC is mediated by the AKT pathway.** (A) Immunoblotting analysis of AKT and phosphorylated-AKT in SPARC-expressing Ishikawa cells. (B) Immunoblotting analysis of SPARC-expressing Ishikawa cells treated with indicated concentrations of the highly selective AKT inhibitor, MK2206. Antibodies against AKT, phosphorylated-AKT (p-AKT, Ser473), CDH2, VIM, FN1 and SPARC were used for immunoblotting. (A, B) Intensity of the bands was quantified using Image J. Values of the protein-of-interest were corrected using the intensity of GAPDH and ACTB bands, respectively. (C) In vitro cell migration of SPARC-expressing Ishikawa cells treated with indicated concentrations of MK2206. Numbers of migrated cells are shown in the top graph. Inhibition ratio of migrated cells compared with numbers of migrated cells at 0 nM is shown in the bottom graph. Ctrl, control; n.s., not significant; *, *P* < 0.05; **, *P* < 0.01. Full-length blot images are presented in Supplementary Fig. 4 and 5 (A, B). The experiments were independently repeated three times and representative data were shown

Next, we studied the impact of SPARC expression in EC cells. Our previous study showed that SPARC expression was absent in Ishikawa cells [24]. We confirmed successful expression and secretion of SPARC in Ishikawa cells by lentivirus-mediated expression (Fig. 2a–c, Supplementary Fig. 2). Forced expression of SPARC in Ishikawa cells induced the protein and mRNA expression of mesenchymal markers CDH2, VIM and FN1 (Fig. 2d and e; Supplementary Fig. 3). In our previous study, we already reported that SPARC-expressing Ishikawa cells expressed remarkably high amounts of FN1 [24]. Consistent with the induction of EMT, cell mobility was enhanced in SPARC-expressing Ishikawa cells (Fig. 2f). SPARC expression in melanoma and non-small cell lung cancer cells was reported to induce EMT mediated by AKT phosphorylation [4, 5]. We found that AKT phosphorylation (Ser473) was induced in Ishikawa cells by SPARC expression (Fig. 3a; Supplementary Fig. 4). As expected, the AKT inhibitor MK2206 canceled the induction of phosphorylated AKT by SPARC expression in a dose-dependent manner (Fig. 3b; Supplementary Fig. 5). Notably, MK2206 also suppressed the expression of CDH2, VIM and FN1 and the cell mobility induced by SPARC expression (Fig. 3b and c; Supplementary Fig. 5). These results suggested that the enhanced EMT and cell mobility by SPARC expression was mediated by AKT signal activation.

### SPARC-expressing EC cells are responsible for the activation of fibroblasts but SPARC alone is not sufficient for the activation

**Fig. 4:**
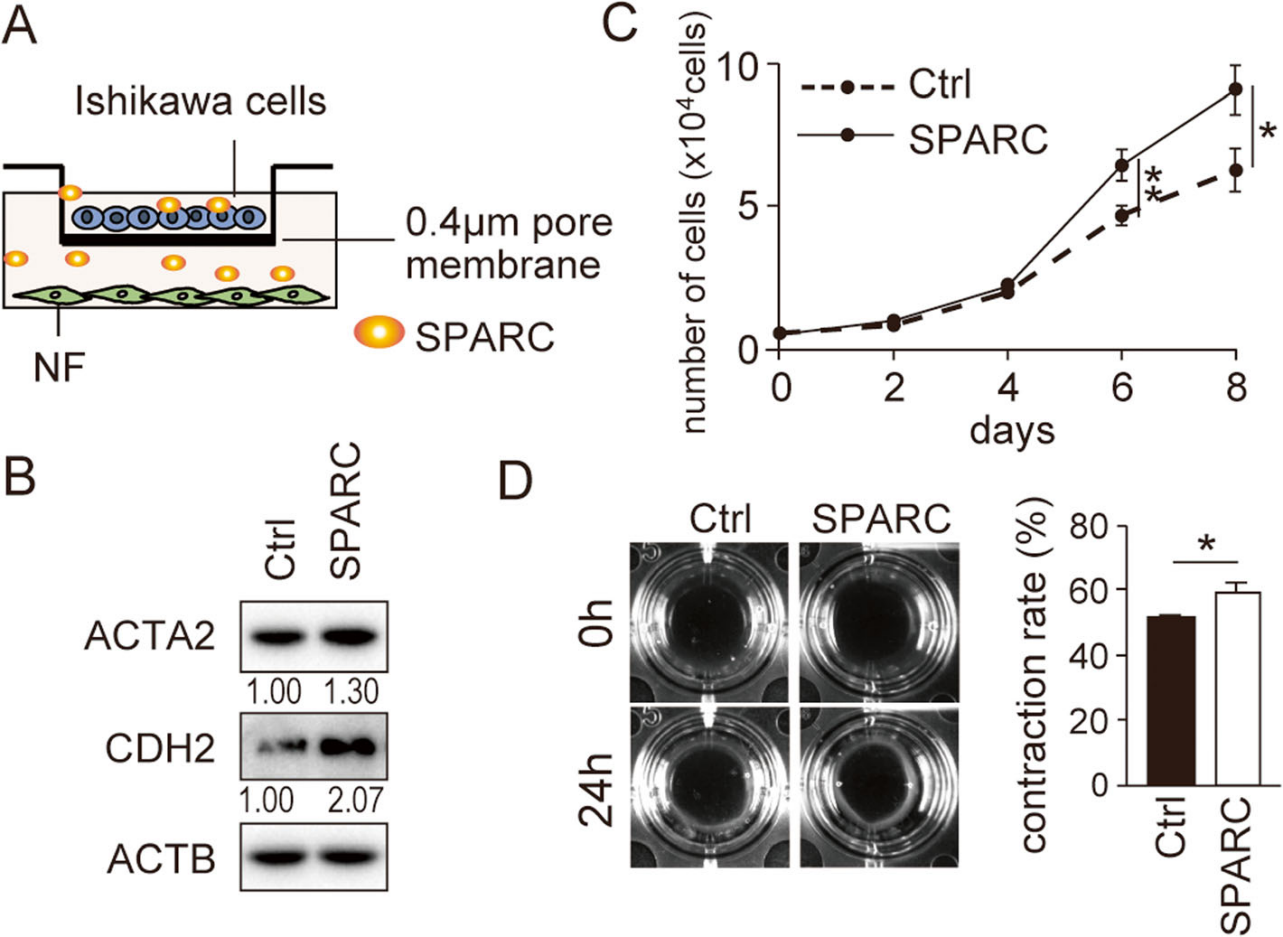
**Co-culture of SPARC-expressing Ishikawa cells activates normal fibroblasts.** (A) Schematic presentation of co-culture experiment of normal fibroblasts (NF) with SPARC-expressing Ishikawa cells. (B) Immunoblotting analysis of ACTA2 and CDH2 in NF co-cultured with SPARC-expressing Ishikawa cells. Intensity of the bands was quantified using Image J. Values of the protein-of-interest were corrected using the intensity of ACTB bands. Full-length blot images are presented in Supplementary Fig. 6. Cell proliferation (C) and in vitro contraction ability (D) of NF were also analyzed. A representative result is shown in the left panels. The right graph shows quantification data of the results. NF, normal fibroblasts; Ctrl, control; *, *P* < 0.05; **, *P* < 0.01. The experiments were repeated using fibroblasts from three independent cases and representative data were shown

**Fig. 5:**
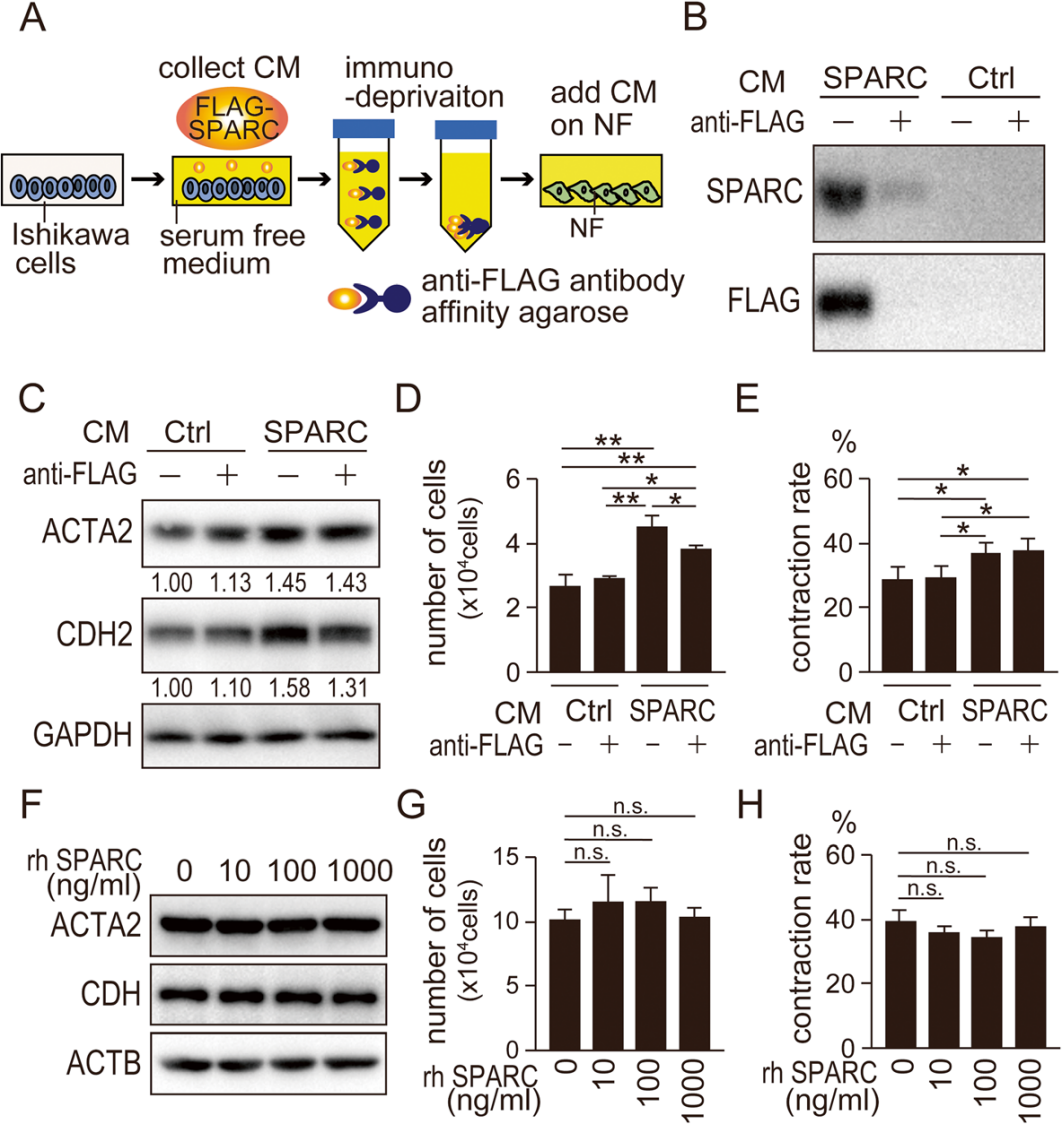
**Deprivation of SPARC in conditioned media from SPARC-expressing Ishikawa cells still activates normal fibroblasts.** (A) Schematic presentation of the production of conditioned media from SPARC-expressing Ishikawa cells immunodepleted of SPARC using anti-FLAG antibody. (B) Successful immunodepletion of SPARC in conditioned media from control and SPARC-expressing Ishikawa cells was confirmed using immunoblotting. (C) Protein expression of ACTA2 and CDH2 in NF cultured in the conditioned media with or without immunodepletion of SPARC. Intensity of the bands was quantified using Image J. Values of the protein-of-interest were corrected using the intensity of GAPDH bands. NF were also analyzed for cell proliferation on day 6 (D) and in vitro contraction (E) in the conditioned media. (F–H) NF were treated with the indicated concentration of recombinant SPARC. Protein expression of ACTA2 and CDH2 (F), cell number on day 6 (G) and in vitro contraction ability (H) were assessed. NF, normal fibroblasts; Ctrl, control; rh SPARC, recombinant human SPARC; CM, conditioned media; n.s., not significant; *, *P* < 0.05; **, *P* < 0.01. Full-length blot images are presented in Supplementary Fig. 7, 8 and 9 (B, C, F). The experiments were independently repeated three times using identical fibroblasts and representative data were shown

We next examined whether SPARC expression in EC cells affected nearby fibroblasts. NF were co-cultured with control or SPARC-expressing Ishikawa cells separated by a membrane (Fig. 4a). SPARC expression in Ishikawa cells enhanced the expression of ACTA2 and CDH2 in the fibroblasts and cell proliferation (Fig. 4b and c; Supplementary Fig. 6). SPARC expression in Ishikawa cells also functionally activated NF; the contraction ability was enhanced in NF co-cultured with SPARC-expressing Ishikawa cells (Fig. 4d). These results suggested that the fibroblasts were activated by a factor secreted from SPARC-expressing EC cells. We thus first examined the impact of SPARC secreted from SPARC-expressing cells on NF. An anti-FLAG antibody was used to immunodeplete FLAG-tagged SPARC from conditioned medium collected from FLAG-SPARC-expressing cells (Fig. 5a). Most of the SPARC was successfully depleted from conditioned media (Fig. 5b; Supplementary Fig. 7). However, the inhibition of ACTA2 and CDH2 by SPARC depletion was only modest (Fig. 5c; Supplementary Fig. 8). The cell proliferation and contraction rate of NF were also not drastically altered by SPARC depletion (Fig. 5d and e). In the further experiments, recombinant SPARC was added to the culture medium of NF. As predicted, recombinant SPARC at the various concentrations examined did not affect the expression of ACTA2 and CDH2, cell proliferation, and contraction ability (Fig. 5f–h; Supplementary Fig. 9). These results suggested that a factor other than SPARC itself secreted from SPARC-expressing EC cells was involved in mediating the effects on NF.

### FN1 is required for activation of fibroblasts by SPARC-expressing EC cells

**Fig. 6:**
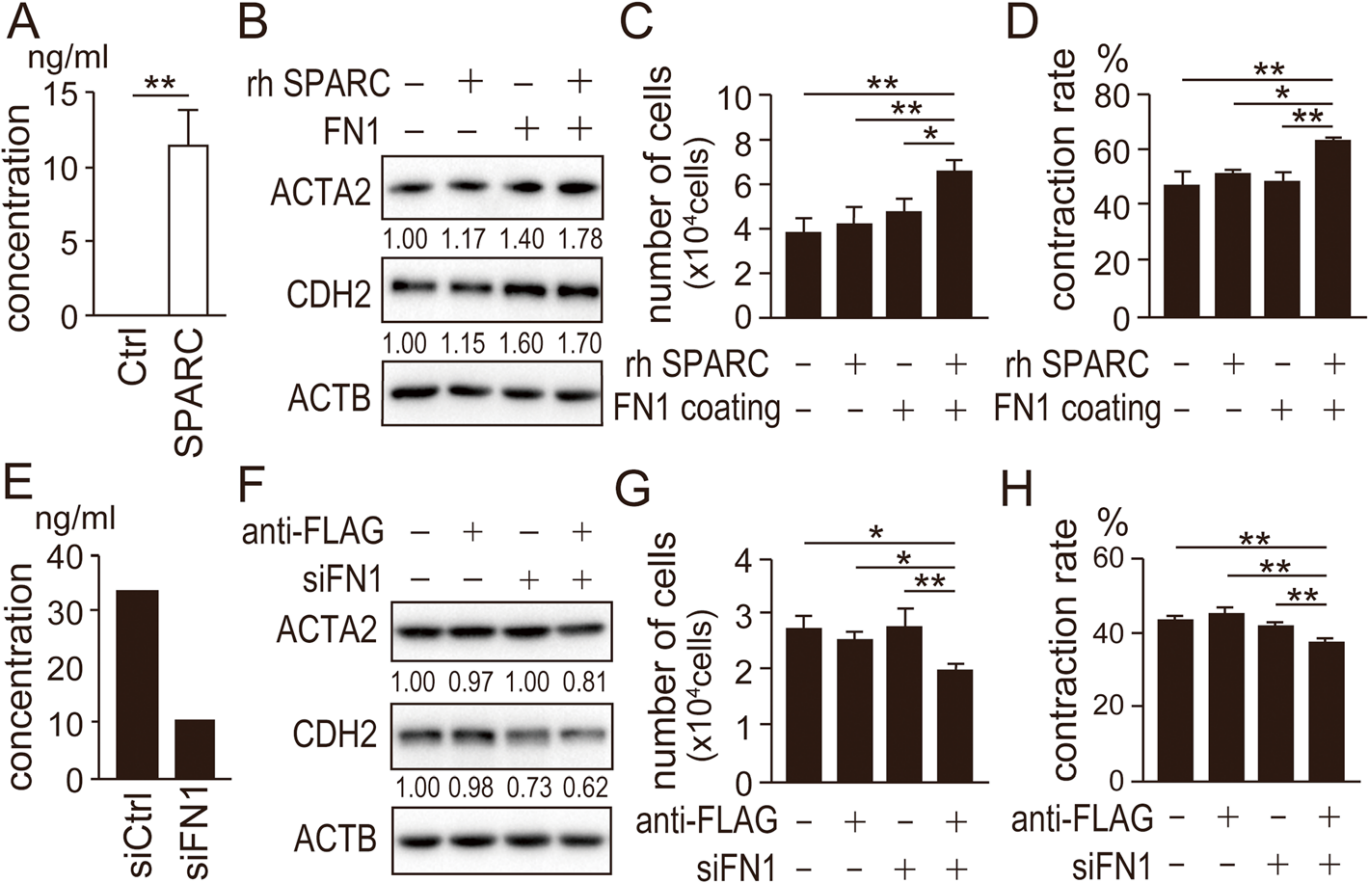
**FN1 secreted from SPARC-expressing Ishikawa cells activates normal fibroblasts.** (A) Amount of FN1 secreted from SPARC-expressing Ishikawa cells was measured by ELISA. (B) Protein levels of ACTA2 and CDH2 in NF cultured on FN1-coated dishes in the presence of recombinant SPARC were analyzed using immunoblotting. (C) NF were also analyzed for cell proliferation on day 6 in the same culture condition as (B). (D) NF cultured in the same condition as (B) were moved to collagen gel for in vitro contraction assays. (E) Successful knockdown of FN1 by siRNA (siFN1) was confirmed using ELISA. (F) Protein levels of ACTA2 and CDH2 in NF cultured in conditioned media from SPARC-expressing Ishikawa cells with siFN1 or immunodepleted of SPARC were analyzed using immunoblotting. NF were also analyzed for cell proliferation on day 6 (G) and in vitro contraction (H) in the conditioned media. (B, F) Intensity of the bands was quantified using Image J. Values of the protein-of-interest were corrected using the intensity of ACTB bands. Ctrl, control; rh SPARC, recombinant human SPARC; siCtrl, control siRNA; *, *P* < 0.05; **, *P* < 0.01. Full-length blot images are presented in Supplementary Fig. 10 and 11 (B, F). The experiments were independently repeated three times using identical fibroblasts and representative data were shown

We next focused on FN1 because abundant FN1 was detected in SPARC-expressing Ishikawa cells compared with control cells (Fig. 2d; Supplementary Fig. 3). We observed higher amounts of FN1 secreted from SPARC-expressing Ishikawa cells compared with control cells (Fig. 6a). We next studied the impact of FN1 on NF. NF on an FN1-coated dish showed elevated expression of ACTA2 and CDH2 (Fig. 6b; Supplementary Fig. 10). Furthermore, addition of recombinant SPARC on NF on the FN1-coated dish enhanced the cell proliferation and contraction ability of NF (Fig. 6c and d). To study the effect of FN1 on NF, we performed knockdown experiments of FN1 in SPARC-expressing Ishikawa cells. Successful siRNA-mediated reduction of FN1 secreted from SPARC-expressing Ishikawa cells was confirmed (Fig. 6e). NF cultured in conditioned media from SPARC-expressing Ishikawa cells in which FN1 was knocked down showed reduced expression of CDH2 (Fig. 6f; Supplementary Fig. 11). In addition, SPARC depletion and FN1 knockdown in SPARC-expressing Ishikawa cells remarkably suppressed the cell proliferation and contraction ability of NF (Fig. 6g and h). These results suggested that FN1 is necessary for the activation of fibroblasts by SPARC in the extracellular matrix. Both FN1 and SPARC abundantly secreted from SPARC-expressing EC cells might be necessary for full activation of fibroblasts.

### SPARC expression in cancer cells is adjacent to FN1 expression in the surrounding stromal tissue

**Fig. 7:**
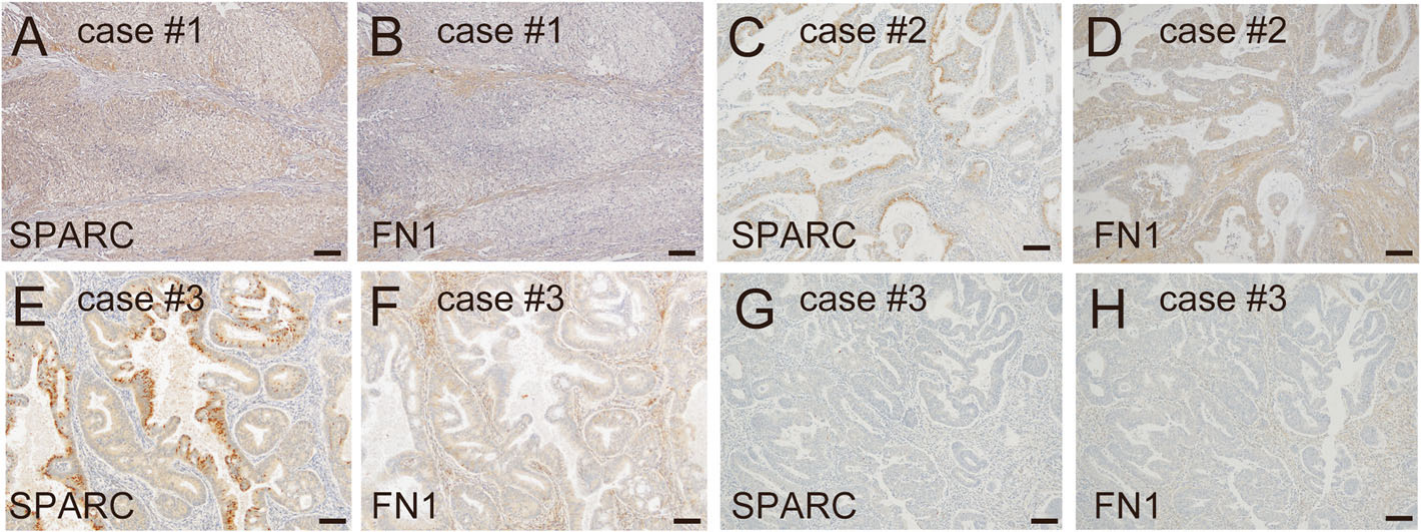
**SPARC expression sites were adjacent to areas with FN1 expression in endometrial cancer tissues.** Three cases of endometrial cancer, (A, B) endometrioid carcinoma grade 3 at stage IA (case #1), (C, D) endometrioid carcinoma grade 1 at stage IA (case #2), and (E–H) endometrioid carcinoma grade 1 at stage IA (case #3) were examined for SPARC (A, C, E, G) and FN1 (B, D, F, H) expression by immunohistochemistry. In the third case, a SPARC-positive area (E) and SPARC-negative area (G) were examined for FN1 expression (F, H). The scale bars indicate 100 μm

Immunohistochemical analysis of EC specimens showed that SPARC expression level was stronger in the cancer cells compared with the stromal area (Fig. 7a–f). This result suggested that SPARC expression and secretion from EC cells affected stromal fibroblasts. Analysis of FN1 expression in the sequential sections showed that SPARC expression in cancer cells was adjacent to FN1 expression in the surrounding stromal tissue (Fig. 7a–f). In some cases, SPARC expression was exclusive in some part of the cancer tissue. The region of cancer tissue positive for SPARC also showed FN1 expression in the stromal area, and the region without SPARC expression also lacked FN1 expression the stromal area (Fig. 7e-h). The above results suggested that FN1 secreted from SPARC-expressing cancer cells pooled in the surrounding stroma to activate the stromal fibroblasts.

## Discussion

Here we showed that SPARC expression in Ishikawa EC cells induced EMT, cell invasion and AKT phosphorylation. The allosteric inhibitor of AKT inhibited EMT and cell migration (Figs. 2 and 3). These results were consistent with previous studies that showed that SPARC induces AKT phosphorylation, which is important for induction of EMT, cell survival, anti-apoptotic activity of SPARC [4, 34, 35].

In addition to the expression of SPARC in cancer cells, its expression in stromal cells also plays a critical role in cancer progression [6, 10, 36, 37]. However, our study showed no remarkable differences in SPARC expression between CAF and NF. In most fibroblasts including NF, the expression level of SPARC was rather high (Fig. 1b and c; Supplementary Fig. 1). In our co-culture assay system of fibroblasts with SPARC-expressing Ishikawa cells, activation of fibroblast-specific gene expression and function was observed (Fig. 4). We observed enhanced proliferation of NF co-cultured with the SPARC-expressing cancer cells in vitro (Fig. 4c). The result may explain why larger amounts of stromal tissues were reported to accompany SPARC-expressing cancer cells in vivo [24, 38, 39]. These results suggested that SPARC expression in fibroblasts was not critical for fibroblast activation, but some factors secreted from SPARC-expressing Ishikawa cells were involved in this activation. Our immunodepletion experiment showed that SPARC itself did not affect fibroblast activation (Fig. 5). Moreover, exogenous addition of recombinant SPARC also did not affect fibroblast activation (Fig. 5). The recombinant SPARC we used was produced in CHO cells and its biological activity was confirmed by osteoblast differentiation of MC3T3 cells at a concentration of 0.5–0.7 μg/mL (PeproTec Inc. Rocky Hill, NJ, USA). Several studies used concentrations of approximately 1.0 μg/mL of recombinant SPARC [9, 40, 41]. We thus applied up to 1.0 μg/mL of recombinant SPARC in our experiments.

Although it was reported that fibroblasts in culture were different from freshly isolated fibroblasts from tissue in gene expression profile, we used fibroblasts passaged minimal times to exclude endothelial cells and epithelial cells [42–44]. Purification of fibroblasts was reported to obtained after at least three passages [45, 46]. NF and CAF at passage 3¬8 were used for experiments as were chosen also in other studies [26–31].

Figure 5d and e showed that immunodepletion of SPARC made only modest changes in fibroblast proliferation and contraction of the fibroblasts. In Fig. 6g and h, immunodepletion of SPARC again made modest changes in the proliferation and contraction. These results were consistent. Next, Fig. 6c and d suggested that FN1 affected the proliferation and contraction only in the presence of SPARC. Considering this result, siFN1 was expected to reduce the proliferation and contraction regardless of SPARC. However, Fig. 6g and h indicated that siFN1 made a difference only in the absence of SPARC. One possible explanation might be that conditioned media from siFN1 transfected cells still produced some amount of FN1 (Fig. 6e). This amount of FN1 might be enough to activate the fibroblast proliferation and contraction only in the presence of SPARC, but not in the absence of SPARC.

In our previous study, we studied the characteristics of EC stem-like cells isolated as side-population (SP) using fluorescence-activated cell sorting. SP fraction of HEC-1 cells expressed more than 7-fold higher amount of FN1 than non-SP fraction of HEC-1 cells [47]. SP HEC-1 cells also overexpressed SPARC compared to non-SP HEC-1 cells [24]. Furthermore, we observed forced expression of SPARC in EC cells induced FN1 expression and secretion (Figs. 2d,e, 3b, 6a ; Supplementary Fig. 3, 5) [24]. These data suggest that abundant FN1 expression in EC stem-like cells depend on overexpression of SPARC.

In support of the in vitro results, our immunohistochemical study showed that SPARC expression sites were adjacent to areas with FN1 expression (Fig. 7). The induction of FN1 by forced expression of SPARC in Ishikawa cells was suggested to mediate AKT activation because an AKT inhibitor suppressed FN1 expression (Fig. 3b; Supplementary Fig. 5). Our results showed that recombinant SPARC induced fibroblast activation only in the presence of FN1 (Figs. 5f-h and 6b-d; Supplementary Fig. 9, 10). SPARC was reported to be critical for FN1-induced ILK activation and actin stress fiber formation in fibroblasts [48]. SPARC expression in cancer cells enhanced their migration and invasion on FN1-coated dishes [49, 50]. Our results also suggested that the cooperation of SPARC and FN1 is critical for full activation of fibroblasts (Fig. 6b-d; Supplementary Fig. 10). A similar conclusion was obtained from our experiments using FN1 knockdown and SPARC depletion in SPARC-expressing Ishikawa cells (Fig. 6f-h; Supplementary Fig. 11). SPARC does not directly support cell attachment and is considered anti-adhesive. SPARC binds to several integral components of the ECM and exhibits an anti-adhesive effect that includes abrogation of focal adhesions and disruption of cell spreading and mobility [51, 52]. SPARC might interact with FN1 to exhibit an anti-adhesive effect.

Activation of fibroblasts is determined not only by gene expression but also by a functional aspect. ECM reorganization and ECM stiffness is reported to be determined by changes in collagen cross-linking, which drives tumor progression through PI3K-AKT activation mediated by integrin receptors [53, 54]. SPARC was reported to interact with integrin [9, 14, 55, 56]. Incorporation of SPARC-integrin-FN1-mediated activation of AKT may be involved in activation of fibroblasts and dominant changes in ECM to help cancer cells to mobilize or invade into adjacent stroma.

The limitation of this study is that the molecular mechanism of SPARC and FN1 for activation of fibroblasts remains to be clarified.

## Conclusions

In conclusion, our study indicated that SPARC induced AKT phosphorylation and EMT in cancer cells, as reported elsewhere [4, 5]. SPARC-expressing endometrial cancer cells activated fibroblasts only in the presence of FN1, which was abundantly secreted from the cancer cells. SPARC and FN1 were suggested to cooperatively activate fibroblasts. These results suggested that a SPARC-fibronectin-mediated activation of fibroblasts might be involved in enhanced mobility and invasion of cancer cells.

## Supplementary Information

**Additional file 1 Table S1.** Age of patients, from which NF were isolated. **Table S2**. Clinical characteristics of samples, from which CAF were isolated.

**Additional file 2 Fig. S1**. Full-length blot images of Fig. 1B.

**Additional file 3 Fig. S2.** Full-length blot images of Fig. 2A.

**Additional file 4 Fig. S3.** Full-length blot images of Fig. 2D.

**Additional file 5 Fig. S4.** Full-length blot images of Fig. 3A.

**Additional file 6 Fig. S5.** Full-length blot images of Fig. 3B.

**Additional file 7 Fig. S6.** Full-length blot images of Fig. 4B.

**Additional file 8 Fig. S7.** Full-length blot images of Fig. 5B.

**Additional file 9 Fig. S8.** Full-length blot images of Fig. 5C.

**Additional file 10 Fig. S9.** Full-length blot images of Fig. 5F.

**Additional file 11 Fig. S10.** Full-length blot images of Fig. 6B.

**Additional file 12 Fig. S11.** Full-length blot images of Fig. 6F.

## Data Availability

All data generated or analyzed during this study are included in this published article.

## Abbreviations

CAF: cancer-associated fibroblasts
EC: endometrial cancer
EMT: epithelial-to-mesenchymal transition
NF: normal fibroblasts
RT-qPCR: reverse transcription quantitative polymerase chain reaction
