# Supplementary material for: Fibronectin mediates activation of stromal fibroblasts by SPARC in endometrial cancer cells
Source: BMC Cancer. 2021 Feb 12;21:156. doi: 10.1186/s12885-021-07875-9 (PMC7881467; doi:10.1186/s12885-021-07875-9)
Supplement: Supplementary file 1 — Additional file 1 Table S1. Age of patients, from which NF were isolated. Table S2. Clinical characteristics of samples, from which CAF were isolated. [file 12885_2021_7875_MOESM1_ESM.docx]

**Table S1**

Age of patient, from which NF were isolated

| NF line no. | Age |
| --- | --- |
| 1 | 31 |
| 2 | 38 |
| 3 | 39 |
| 4 | 32 |
| 5 | 42 |
| 6 | 34 |

NF, normal fibroblasts

**Table S2**

Clinical characteristics of samples, from which CAF were isolated

| CAF line no. | Age | Histological subtype | Staging |
| --- | --- | --- | --- |
| 1 | 76 | Endometrioid carcinoma, grade 1 | II |
| 2 | 40 | Endometrioid carcinoma, grade 1 | IA |
| 3 | 79 | Serous carcinoma | IVB |
| 4 | 65 | Endometrioid carcinoma, grade 1 | IB |
| 5 | 54 | Serous carcinoma | IIIC |
| 6 | 54 | Endometrioid carcinoma, grade 2 | IA |
| 7 | 35 | Endometrioid carcinoma, grade 1 | IA |

CAF, cancer-associated fibroblasts
