# Supplementary figures and images for: Fibronectin mediates activation of stromal fibroblasts by SPARC in endometrial cancer cells
Source: BMC Cancer. 2021 Feb 12;21:156. doi: 10.1186/s12885-021-07875-9 (PMC7881467; doi:10.1186/s12885-021-07875-9)

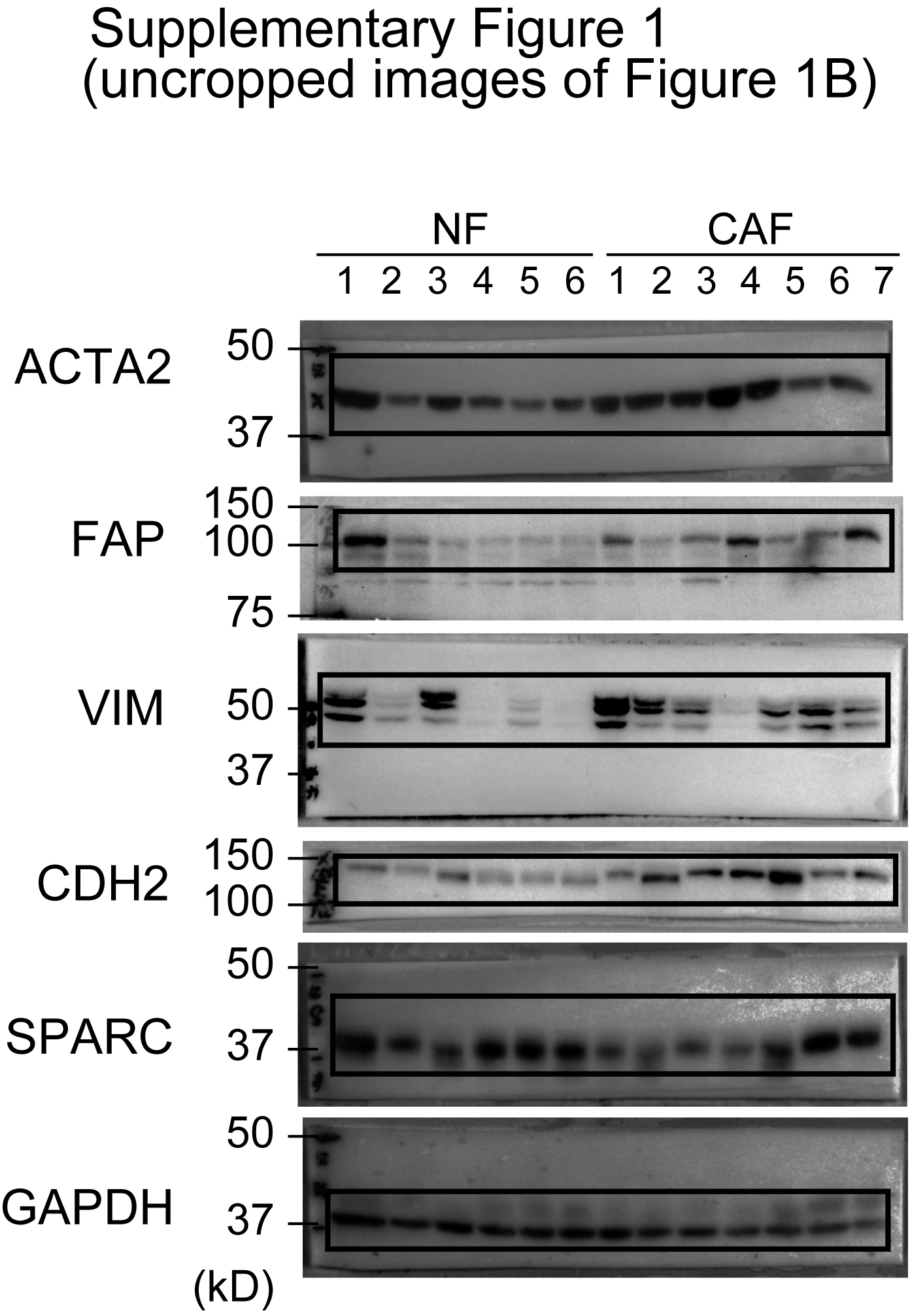

Supplement: Supplementary file 2 — Additional file 2 Fig. S1. Full-length blot images of Fig. 1B. [file 12885_2021_7875_MOESM2_ESM.tif]

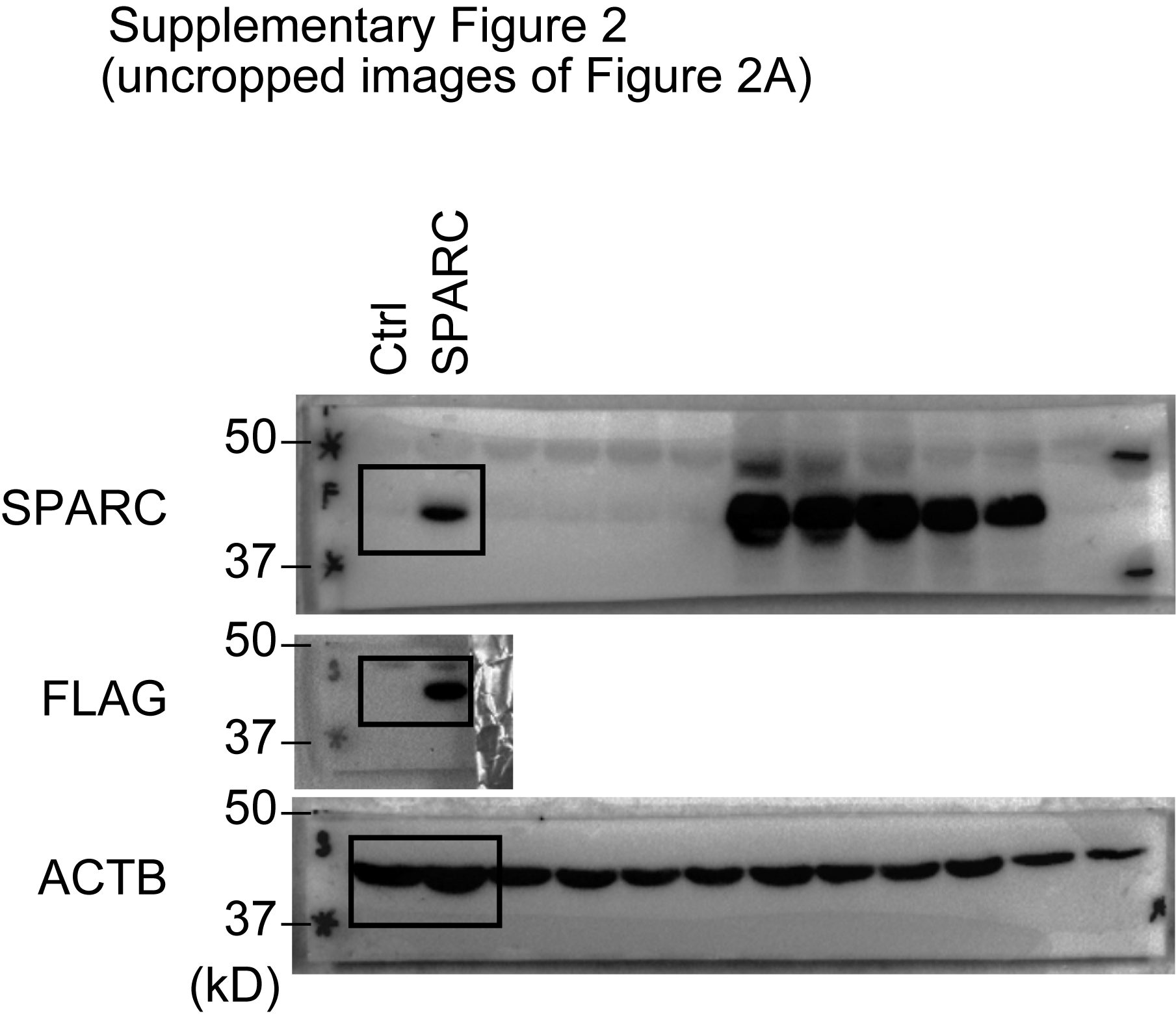

Supplement: Supplementary file 3 — Additional file 3 Fig. S2. Full-length blot images of Fig. 2A. [file 12885_2021_7875_MOESM3_ESM.tif]

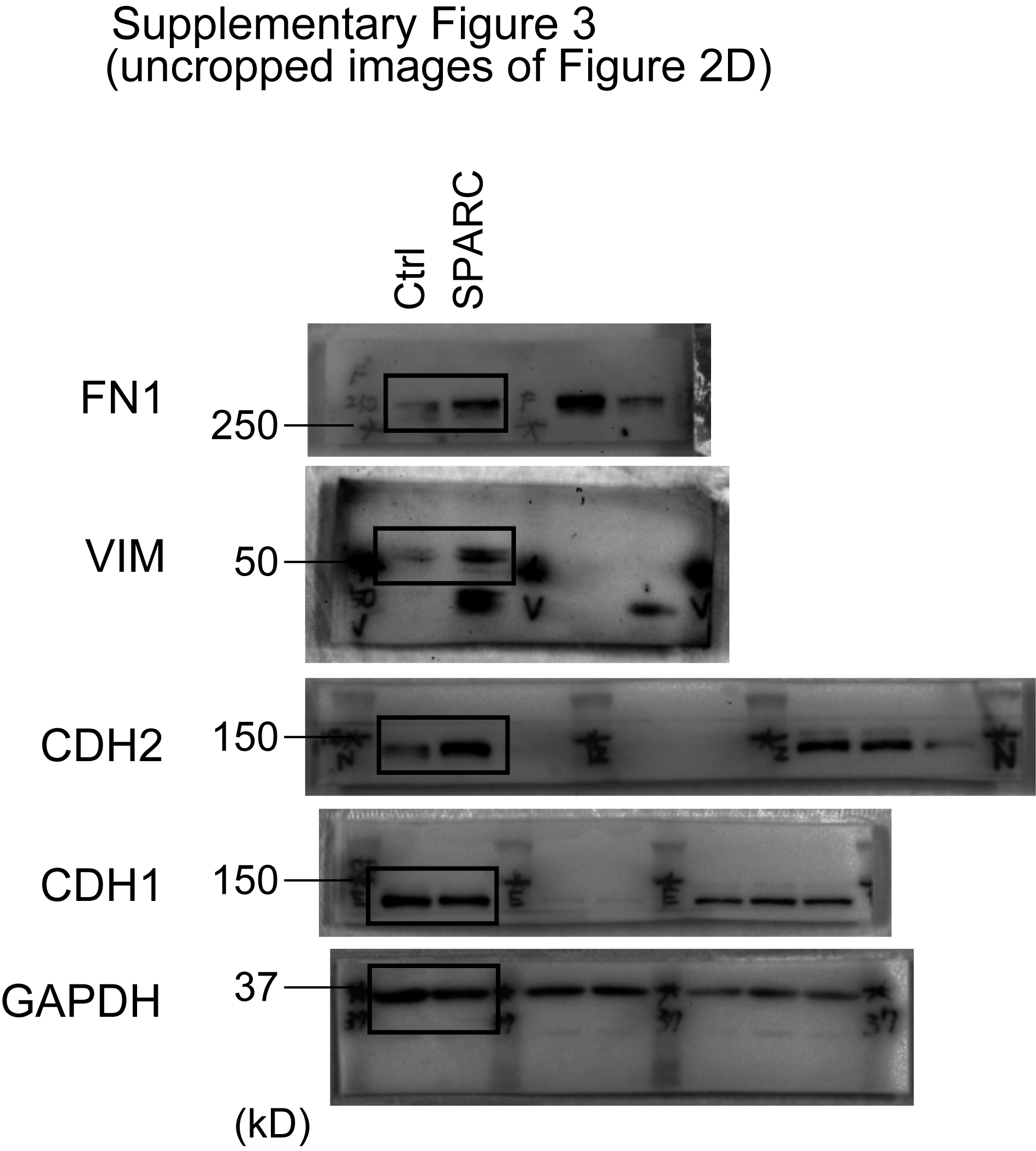

Supplement: Supplementary file 4 — Additional file 4 Fig. S3. Full-length blot images of Fig. 2D. [file 12885_2021_7875_MOESM4_ESM.tif]

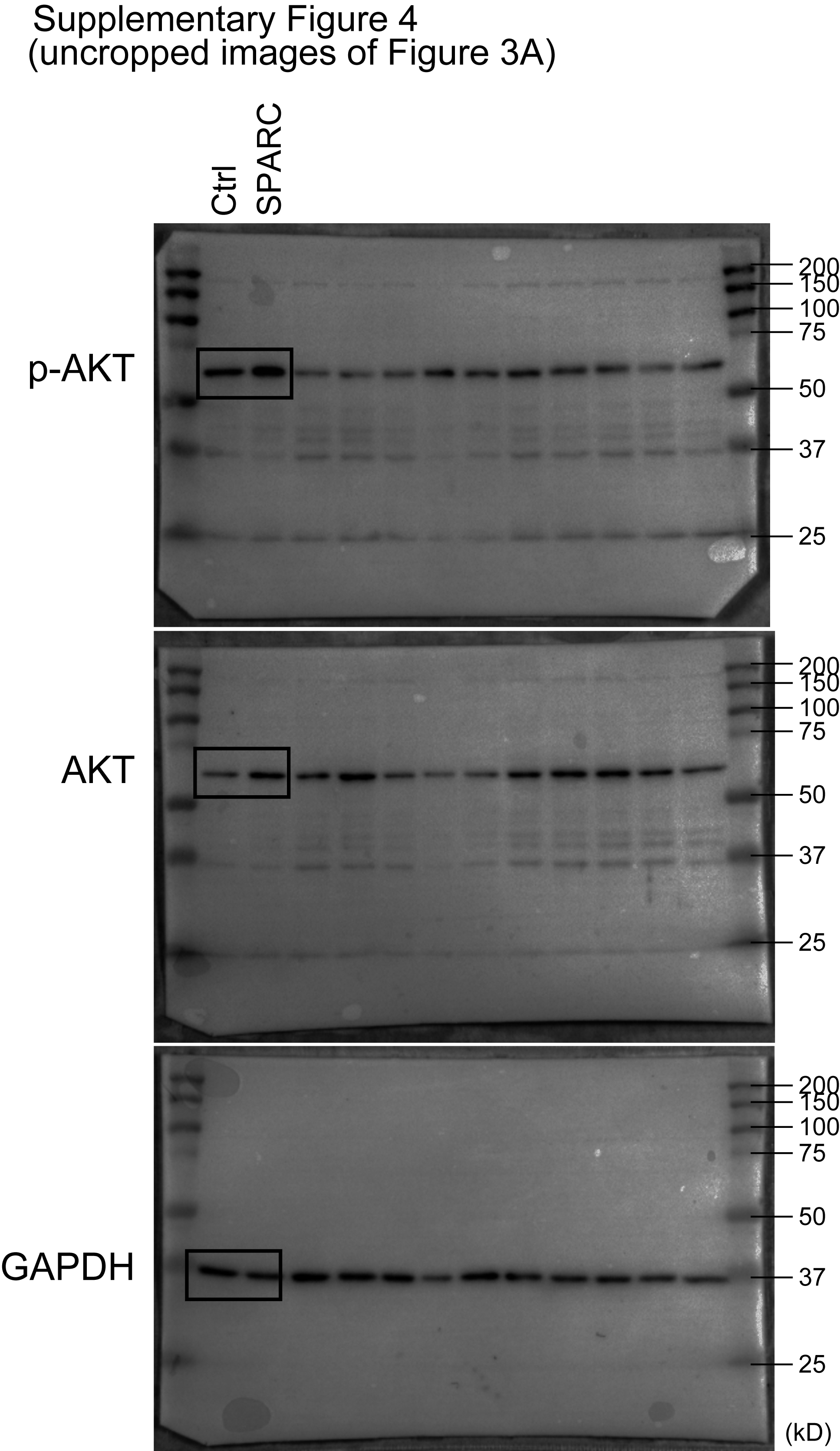

Supplement: Supplementary file 5 — Additional file 5 Fig. S4. Full-length blot images of Fig. 3A. [file 12885_2021_7875_MOESM5_ESM.tif]

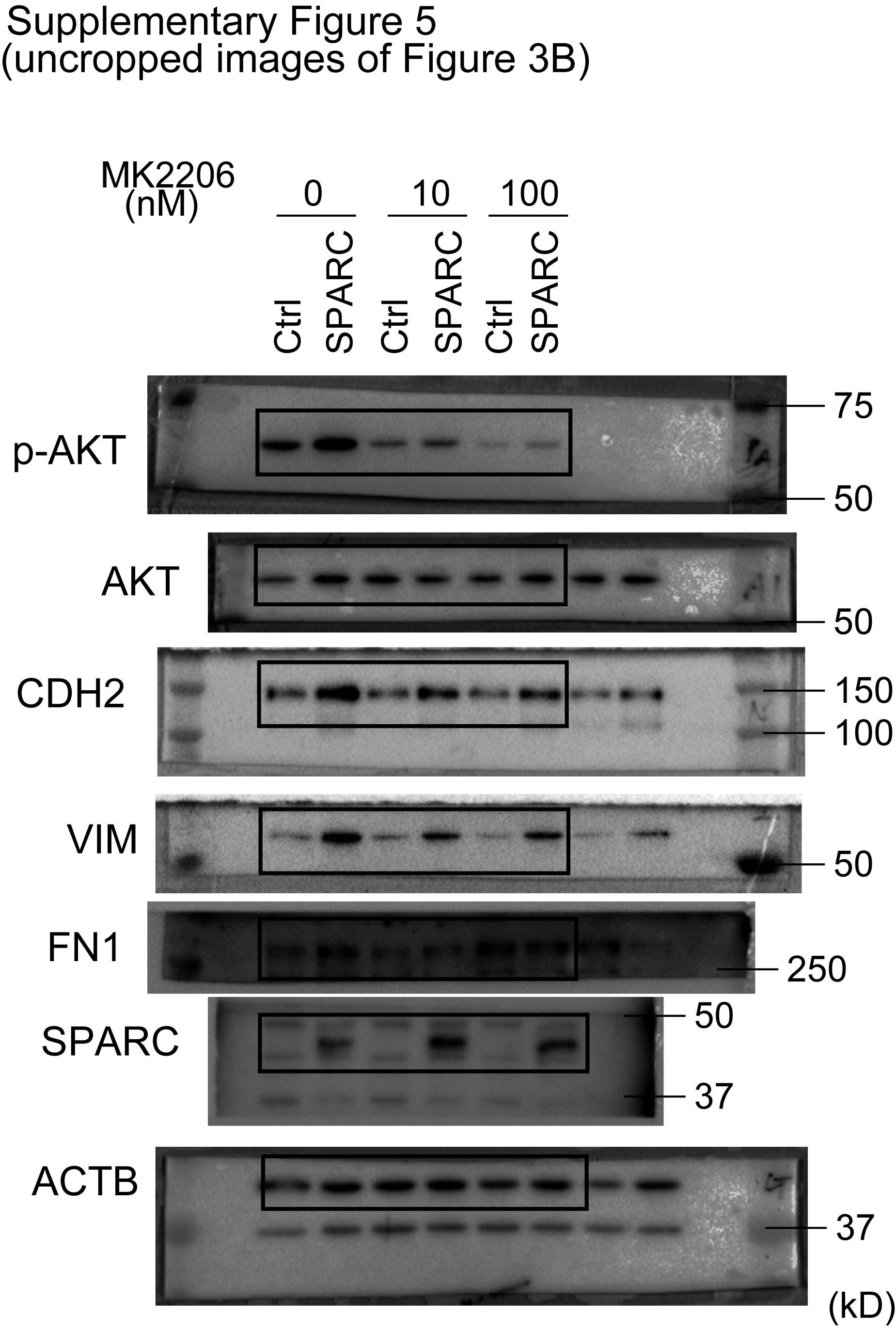

Supplement: Supplementary file 6 — Additional file 6 Fig. S5. Full-length blot images of Fig. 3B. [file 12885_2021_7875_MOESM6_ESM.tif]

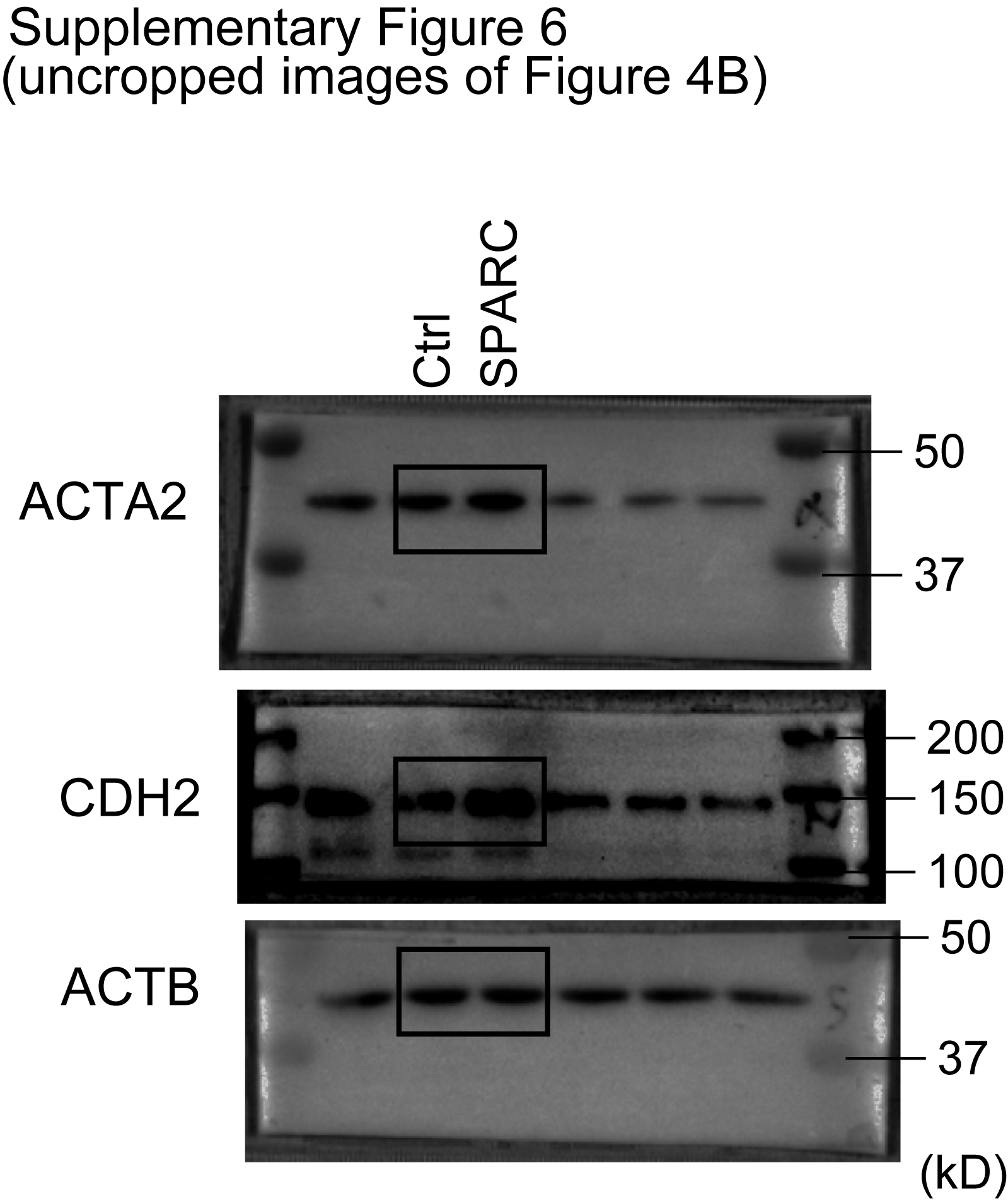

Supplement: Supplementary file 7 — Additional file 7 Fig. S6. Full-length blot images of Fig. 4B. [file 12885_2021_7875_MOESM7_ESM.tif]

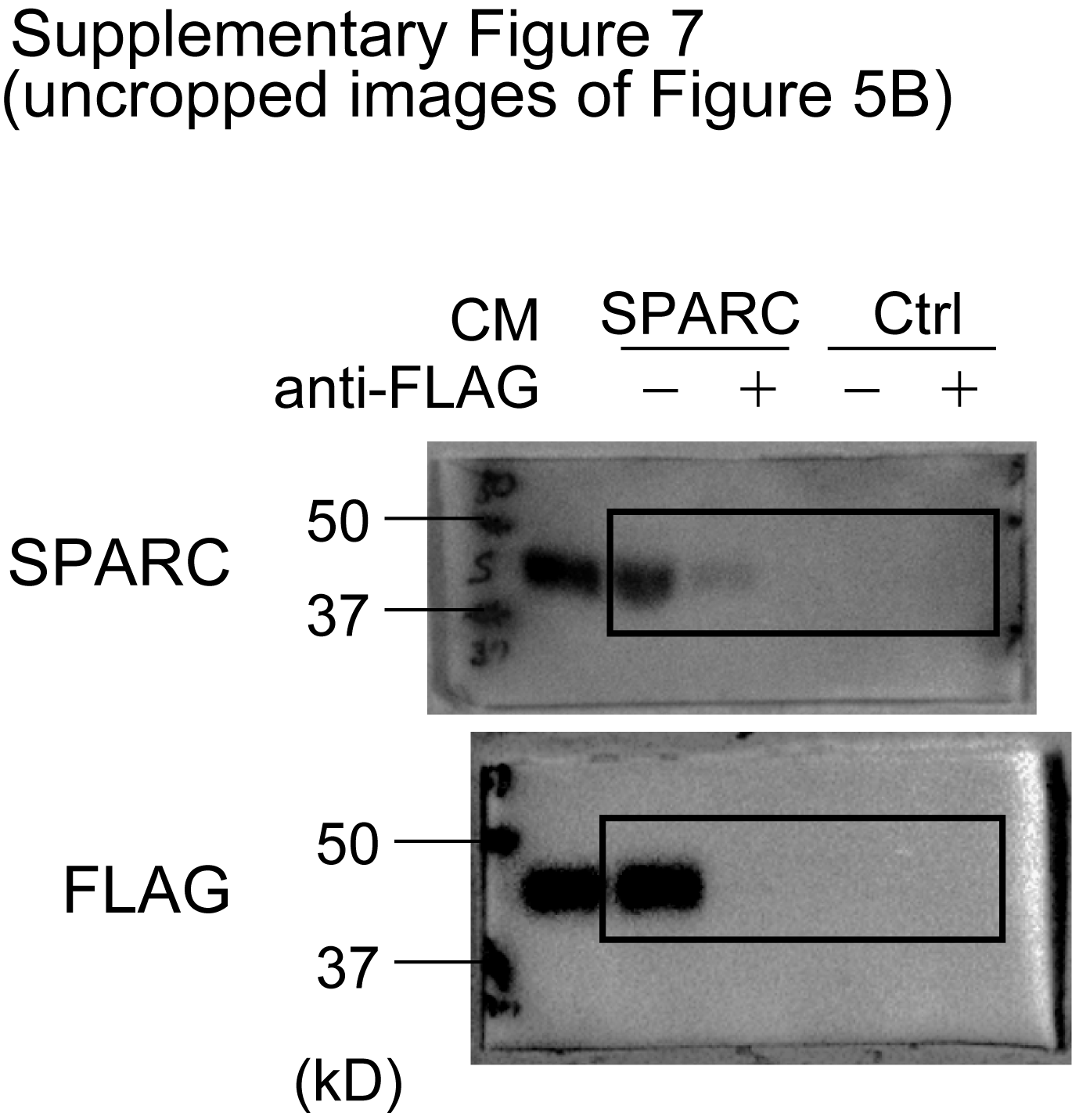

Supplement: Supplementary file 8 — Additional file 8 Fig. S7. Full-length blot images of Fig. 5B. [file 12885_2021_7875_MOESM8_ESM.tif]

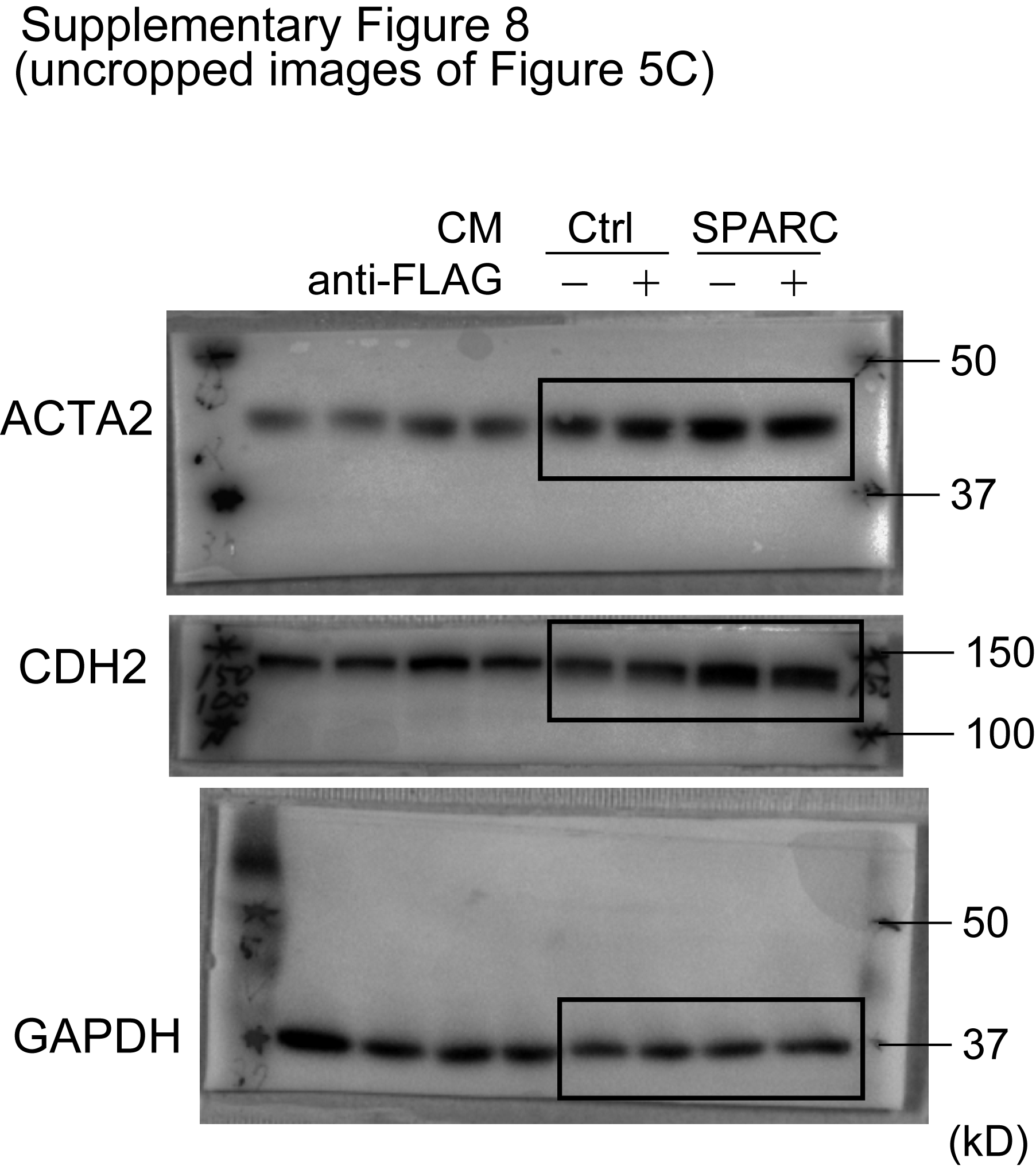

Supplement: Supplementary file 9 — Additional file 9 Fig. S8. Full-length blot images of Fig. 5C. [file 12885_2021_7875_MOESM9_ESM.tif]

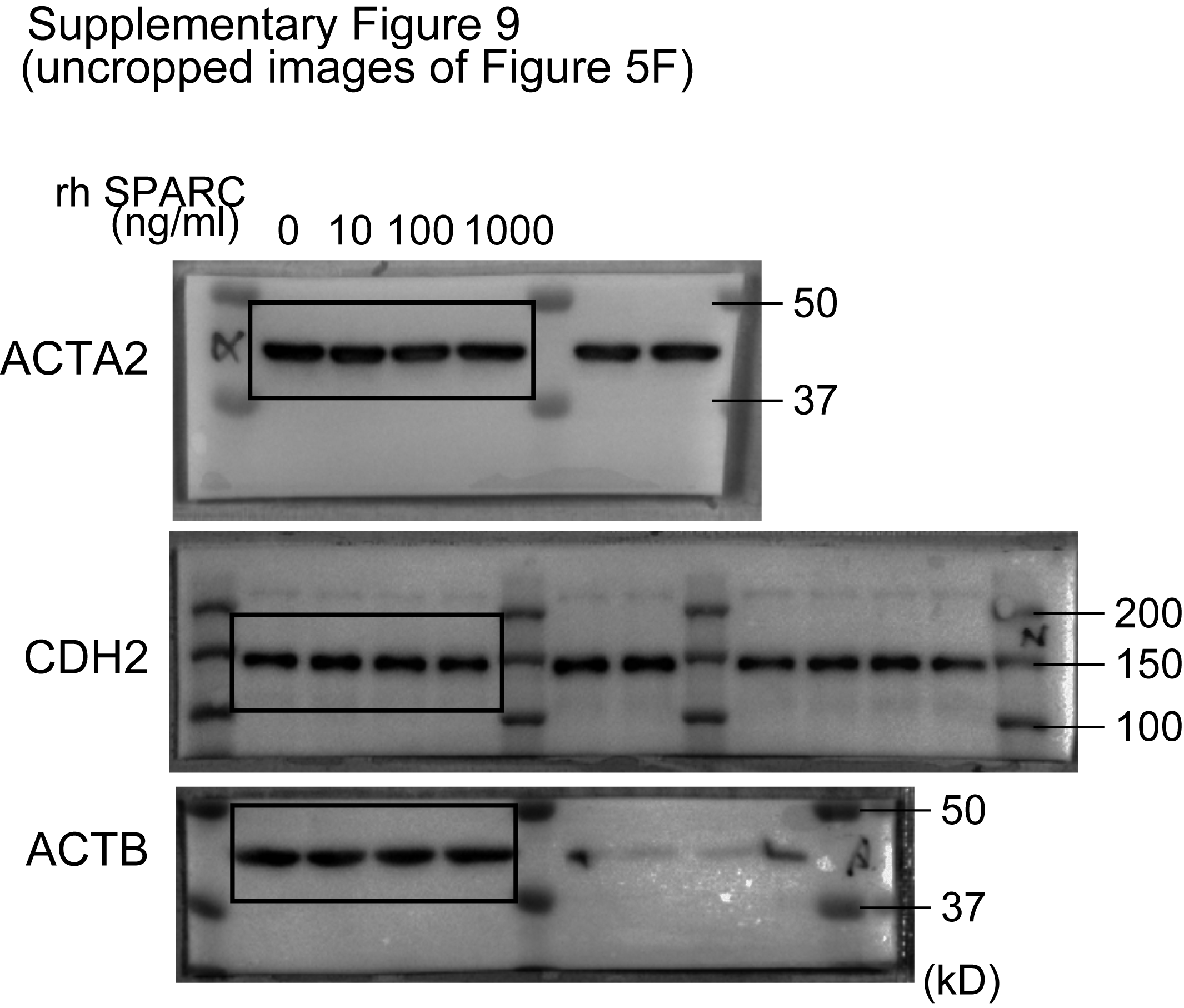

Supplement: Supplementary file 10 — Additional file 10 Fig. S9. Full-length blot images of Fig. 5F. [file 12885_2021_7875_MOESM10_ESM.tif]

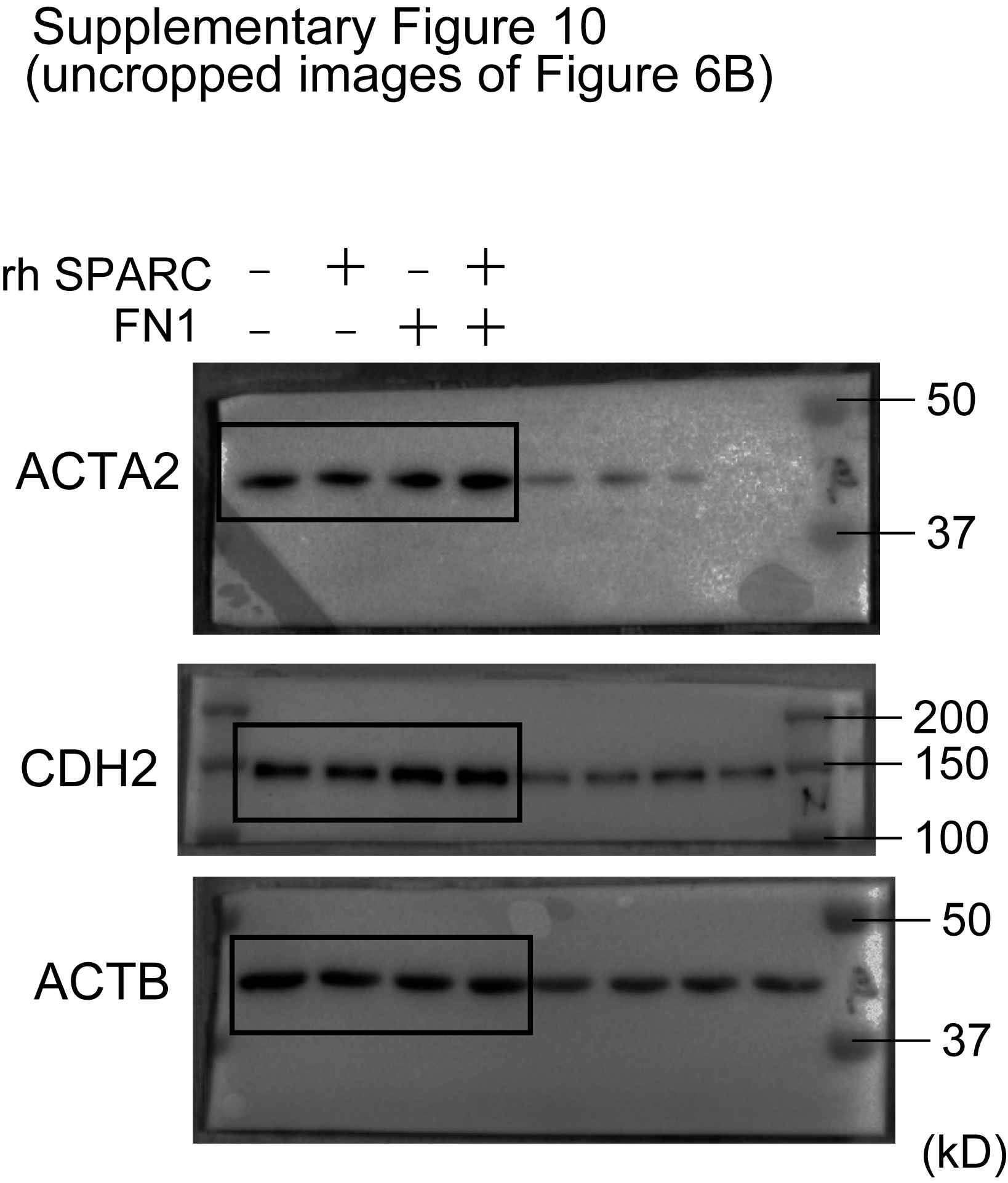

Supplement: Supplementary file 11 — Additional file 11 Fig. S10. Full-length blot images of Fig. 6B. [file 12885_2021_7875_MOESM11_ESM.tif]

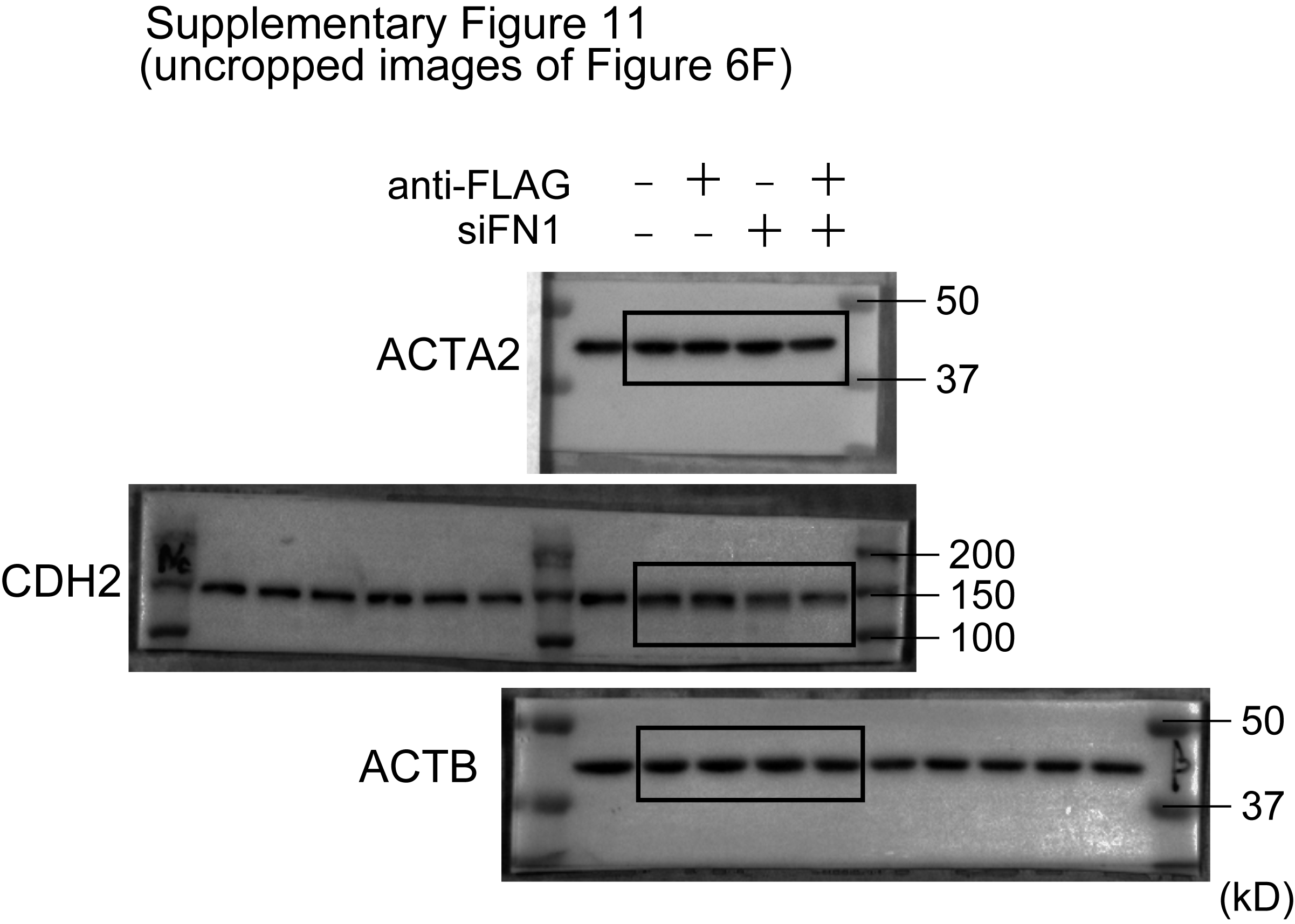

Supplement: Supplementary file 12 — Additional file 12 Fig. S11. Full-length blot images of Fig. 6F. [file 12885_2021_7875_MOESM12_ESM.tif]
